# Supplementary material for: Deletion of wfs1 Impairs Oligodendrocyte Precursor Cells Dorsal Distribution and Myelination Through the wfs1-hmgcs1 Axis in Zebrafish
Source: Biology (Basel). 2026 Aug 16;15(16):1402. doi: 10.3390/biology15161402 (PMC13510151; doi:10.3390/biology15161402)
Supplement: Supplementary file 1 [file biology-15-01402-s001.zip › File S1-Transcriptome sequencing data and differentially expressed genes (DEGs) in wfs1-- zebrafish at 5 dpf/2.KEGG/wfs1KOvsWT/GSEA/WT/AUTOPHAGY___OTHER/AUTOPHAGY___OTHER(DRE04136).html]

Details for gene set AUTOPHAGY\_\_\_OTHER(DRE04136)[GSEA]

|  || Dataset | deg.sample |
| Phenotype | sample.cls |
| Upregulated in class | WT |
| GeneSet | AUTOPHAGY\_\_\_OTHER(DRE04136) |
| Enrichment Score (ES) | -0.3017799 |
| Normalized Enrichment Score (NES) | -1.2514977 |
| Nominal p-value | 0.19483101 |
| FDR q-value | 0.51898044 |
| FWER p-Value | 0.987 |
Table: GSEA Results Summary

  

Fig 1: Enrichment plot: AUTOPHAGY\_\_\_OTHER(DRE04136)      
 Profile of the Running ES Score & Positions of GeneSet Members on the Rank Ordered List

  

| PROBE | DESCRIPTION (from dataset) | GENE SYMBOL | GENE\_TITLE | RANK IN GENE LIST | RANK METRIC SCORE | RUNNING ES | CORE ENRICHMENT || 1 | 326974\_1 | gabarapa |  |  | 1391 | 1.732 | 0.0295 | No |
| 2 | 795933 | atg4da |  |  | 2830 | 1.076 | 0.0299 | No |
| 3 | 100000242\_1 | zgc:92606 |  |  | 3071 | 1.020 | 0.0654 | No |
| 4 | 406582 | ppp2cb |  |  | 3436 | 0.944 | 0.0939 | No |
| 5 | 406843\_1 | igbp1 |  |  | 4369 | 0.766 | 0.0970 | No |
| 6 | 792689 | mlst8 |  |  | 4430 | 0.756 | 0.1271 | No |
| 7 | 101883461\_1 | si:ch73-45o6.2 |  |  | 6559 | 0.532 | 0.0828 | No |
| 8 | 448859 | atg4b |  |  | 7053 | 0.487 | 0.0879 | No |
| 9 | 101884161 | LOC101884161 |  |  | 7207 | 0.473 | 0.1031 | No |
| 10 | 415193 | atg4c |  |  | 8047 | 0.419 | 0.0945 | No |
| 11 | 791223 | atg9b |  |  | 8583 | 0.396 | 0.0944 | No |
| 12 | 560455\_1 | rptor |  |  | 8701 | 0.388 | 0.1072 | No |
| 13 | 324254 | mtor |  |  | 9164 | 0.355 | 0.1077 | No |
| 14 | 100329702 | ulk2 |  |  | 9563 | 0.329 | 0.1091 | No |
| 15 | 793200 | gabarapb |  |  | 9960 | 0.306 | 0.1096 | No |
| 16 | 100332293 | LOC100332293 |  |  | 10824 | 0.254 | 0.0932 | No |
| 17 | 570965\_1 | atg12 |  |  | 11314 | 0.226 | 0.0875 | No |
| 18 | 336632 | atg3 |  |  | 12708 | 0.154 | 0.0502 | No |
| 19 | 406582\_1 | ppp2cb |  |  | 12771 | 0.151 | 0.0547 | No |
| 20 | 550552 | atg16l1 |  |  | 13427 | 0.119 | 0.0391 | No |
| 21 | 550585 | ppp2ca |  |  | 14235 | 0.081 | 0.0172 | No |
| 22 | 100009663 | atg9a |  |  | 14551 | 0.067 | 0.0102 | No |
| 23 | 494180 | atg5 |  |  | 14638 | 0.063 | 0.0101 | No |
| 24 | 403036 | gabarapl2 |  |  | 15513 | 0.022 | -0.0164 | No |
| 25 | 564088 | pik3r4 |  |  | 17575 | -0.057 | -0.0787 | No |
| 26 | 101883461 | si:ch73-45o6.2 |  |  | 18697 | -0.108 | -0.1093 | No |
| 27 | 393885 | zgc:56064 |  |  | 18701 | -0.108 | -0.1048 | No |
| 28 | 393362 | wipi1 |  |  | 19013 | -0.122 | -0.1094 | No |
| 29 | 567680 | atg101 |  |  | 19792 | -0.157 | -0.1272 | No |
| 30 | 437002 | wipi2 |  |  | 20125 | -0.172 | -0.1304 | No |
| 31 | 393405 | atg13 |  |  | 22362 | -0.287 | -0.1885 | No |
| 32 | 324254\_1 | mtor |  |  | 22584 | -0.301 | -0.1827 | No |
| 33 | 393846 | becn1 |  |  | 23266 | -0.345 | -0.1895 | No |
| 34 | 548346 | pik3c3 |  |  | 24150 | -0.403 | -0.2002 | No |
| 35 | 100333016 | atg7 |  |  | 25106 | -0.454 | -0.2110 | No |
| 36 | 641330 | atg10 |  |  | 25462 | -0.481 | -0.2018 | No |
| 37 | 554140 | atg4a |  |  | 27096 | -0.639 | -0.2260 | Yes |
| 38 | 560455 | rptor |  |  | 27664 | -0.695 | -0.2145 | Yes |
| 39 | 326974 | gabarapa |  |  | 27990 | -0.740 | -0.1934 | Yes |
| 40 | 792689\_1 | mlst8 |  |  | 31444 | -2.758 | -0.1852 | Yes |
| 41 | 100000242 | zgc:92606 |  |  | 31809 | -4.711 | 0.0025 | Yes |
Table: GSEA details [plain text format]

  

Fig 2: AUTOPHAGY\_\_\_OTHER(DRE04136)      
 Blue-Pink O' Gram in the Space of the Analyzed GeneSet

  

Fig 3: AUTOPHAGY\_\_\_OTHER(DRE04136): Random ES distribution      
 Gene set null distribution of ES for **AUTOPHAGY\_\_\_OTHER(DRE04136)**
